# Patterns and potential drivers of intraspecific variability in the body C, N, and P composition of a terrestrial consumer, the snowshoe hare (*Lepus americanus*)

**DOI:** 10.1002/ece3.5880

**Published:** 2019-12-08

**Authors:** Matteo Rizzuto, Shawn J. Leroux, Eric Vander Wal, Yolanda F. Wiersma, Travis R. Heckford, Juliana Balluffi‐Fry

**Affiliations:** ^1^ Department of Biology Memorial University of Newfoundland St. John's NF Canada

**Keywords:** boreal forest, carbon, ecological stoichiometry, ecosystem ecology, herbivore, intraspecific variability, nitrogen, phosphorus

## Abstract

1. Intraspecific variability in ecological traits is widespread in nature. Recent evidence, mostly from aquatic ecosystems, shows individuals differing at the most fundamental level, that of their chemical composition. Age, sex, or body size and condition may be key drivers of intraspecific variability in the body concentrations of carbon (C), nitrogen (N), and phosphorus (P). However, we still have a rudimentary understanding of the patterns and drivers of intraspecific variability in chemical composition of terrestrial consumers, particularly vertebrates.
2. Here, we investigate the elemental composition of the snowshoe hare *Lepus americanus*. Based on snowshoe hare ecology, we predicted older, larger individuals to have higher concentration of N or P and lower C content compared with younger, smaller individuals. We also predicted females to have higher concentrations of N, P, and lower C than males due to the higher reproductive costs they incur. Finally, we predicted that individuals in better body condition would have higher N and P than those in worse condition, irrespective of age.
3. We obtained C, N, and P concentrations and ratios from a sample of 50 snowshoe hares. We then used general linear models to test our predictions on the relationship between age, sex, body size or condition and stoichiometric variability in hares.
4. We found considerable variation in C, N, and P stoichiometry within our sample. Contrary to our predictions, we found weak evidence of N content decreasing with age. As well, sex appeared to have no relationship with hare body elemental composition. Conversely, as expected, P content increased with body size and condition. Finally, we found no relationship between variability in C content and any of our predictor variables.
5. Snowshoe hare stoichiometry does not appear to vary with individual age, sex, body size, or condition. However, the weak relationship between body N concentration and age may suggest varying nutritional requirements of individuals at different ages. Conversely, body P's weak relationship to body size and condition appears in line with this limiting element's importance in terrestrial ecosystems. Snowshoe hares are keystone herbivores in the boreal forest of North America, and the substantial stoichiometric variability we find in our sample could have important implications for nutrient dynamics, in both boreal and adjacent ecosystems.

## Graphical abstract

We investigate variability in whole‐body carbon, nitrogen, and phosphorus concentrations and ratios of snowshoe hares. We test whether age, sex, and body size may serve as predictors of this variability. We expected higher P and N concentrations (a) in younger individuals, as well as (b) in females, and (c) in individuals in better condition. While we found no substantial support for age, sex, or body size as predictors of this variation, we nonetheless observe considerable stoichiometric variability in our sample, which may have important implications for nutrient dynamics in boreal and adjacent ecosystems.

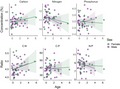

## 1 INTRODUCTION

The elemental composition of an organism is an important ecological trait subject to variation within and across species (Jeyasingh, Cothran, & Tobler, 2014; Leal, Seehausen, & Matthews, 2017). Primary producers (e.g., plants, algae), owing to the presence of dedicated storage structures in their cells, are plastic in their elemental composition (Borer et al., 2013; Sterner & Elser, 2002): individual stoichiometric variability can at times be as large as that found among different genotypes (Ågren & Weih, 2012). Marine phytoplankton and terrestrial plants show large variability in their carbon (C), nitrogen (N), and phosphorus (P) concentrations, at both large (Martiny et al., 2013; Sardans et al., 2016) and small spatio‐temporal extents (Rivas‐Ubach, Sardans, Perez‐Trujillo, Estiarte, & Penuelas, 2012). Conversely, intraspecific variability in the chemical composition of consumers is generally considered smaller than variability observed in autotrophs, due to strict homeostasis requirements—particularly for terrestrial consumers (Elser et al., 2007; Leroux & Schmitz, 2015; Sterner & Elser, 2002). However, studies of invertebrates (González, Fariña, Kay, Pinto, & Marquet, 2011) or aquatic consumers (e.g., fish; Ebel, Leroux, Robertson, & Dempson, 2015; Ebel, Leroux, Robertson, & Dempson, 2016) recently challenged this view, showing evidence of considerable intraspecific stoichiometric variability in these species. For terrestrial vertebrates, much research has focused on their nutritional body composition (Hewison et al., 1996), differential use of chemical elements among conspecifics (Atwood & Weeks, 2002), or body condition (Peig & Green, 2010). We know little, however, about their organismal elemental composition, how it interacts with other ecological traits, and whether it varies among individuals. Given the very different patterns of energy and nutrient flows in aquatic and terrestrial ecosystems, which are driven primarily by the greater resource investments in structural support structures by terrestrial autotrophs (Shurin, Gruner, & Hillebrand, 2006), we may expect differences in vertebrate consumer body composition in different ecosystems. Knowledge of the patterns and drivers of terrestrial vertebrate body elemental composition may shed light on how they shape a species' ecological niche (González et al., 2018; González, Dézerald, Marquet, Romero, & Srivastava, 2017; Peñuelas et al., 2019). Further, it may improve our ability to predict the relationship between consumers and ecosystem processes (e.g., carbon cycling; Schmitz et al., 2014).

Herbivores have the potential to exert top‐down control on primary producers and can also affect their predators' ecology (Leroux & Schmitz, 2015). They rely on resources whose organismal stoichiometry is markedly different from their own: terrestrial plants and algae are rich in C‐heavy structural molecules, while herbivores rely on N and P to fuel their growth (Fagan et al., 2002; Sterner & Elser, 2002). This mismatch, especially evident in terrestrial food webs, creates a strong bottleneck to nutrient flow in ecosystems (Boersma et al., 2008; Leroux & Schmitz, 2015). As such, investigating the drivers of intraspecific variability in elemental composition of terrestrial herbivores can help shed light on both trophic dynamics and ecosystem processes, such as nutrient cycling (Leroux & Schmitz, 2015; Schmitz et al., 2018; Sterner & Elser, 2002). Previous studies showed that consumers' elemental composition may vary under the effect of a wide range of variables and, in particular, as a function of an individual's age, sex, or body size and condition (Ebel et al., 2015; El‐Sabaawi et al., 2014; El‐Sabaawi, Zandonà, et al., 2012). Here, we investigate how these three variables influence the C, N, P body composition of a terrestrial consumer common across North America's boreal forest, the snowshoe hare *Lepus americanus*. We focus on C, N, and P, as these are three of the most commonly studied and important elements for an organism (Sterner & Elser, 2002; but see Jeyasingh et al., 2014). Owing to the strong nutrient limitation of boreal ecosystems (Pastor, Cohen, & Hobbs, 2006), their unique ecology (Feldhamer, Thompson, & Chapman, 2003), and their role as keystone herbivores in the boreal forest (Krebs, Boonstra, & Boutin, 2018), snowshoe hares are well‐suited to address these questions.

Organismal elemental content can vary throughout an individual's life. For instance, early life stages of *Daphnia lumholtzi* show higher concentrations of P and lower N:P than older ones, that appear to more strongly influence their growth rate than their body size (Main, Dobberfuhl, & Elser, 1997). Evidence shows this pattern holds true among freshwater insects as well (Back & King, 2013). Furthermore, similar intraspecific differences in elemental concentrations between life stages also exist among vertebrates (El‐Sabaawi, Kohler, et al., 2012; El‐Sabaawi et al., 2014; El‐Sabaawi, Zandonà, et al., 2012). At times, this ontogenic variation in elemental composition of conspecifics is as large as that found among different genera (e.g., *Pimephales promelas* and *Cyprinodon variegatus*; Boros, Sály, & Vanni, 2015). This allows for describing life stage‐specific elemental signatures, as recently done for pre‐ and post‐spawn adult Atlantic salmon *Salmo salar* during their annual spawning migration up‐ and downstream, respectively (Ebel et al., 2016). Similarly, the transition from newborn to adult in mammals involves a wide range of developmental changes, for example, skeletal development and gonadal maturation, that could influence the elemental requirements and composition of an individual as it grows. For instance, Sterner and Elser (2002) hypothesize that, as bone tissue should contain most of its P reserves, a vertebrate's P content should increase with age given skeletal growth. Snowshoe hare develop quickly from newborn to adult but live in a strongly nutrient‐limited environment: the trade‐offs they face in acquiring necessary nutrients throughout their lifetime makes them well‐suited to investigate how age affects vertebrate intraspecific stoichiometry.

In a similar way, sex could affect relative content of key elements, due to differences in reproductive strategies and roles between males and females. Female mayflies, for instance, tend to have higher %P than males and slower %P decline with age (Back & King, 2013). Among vertebrates, three‐spined stickleback *Gasterosteus aculeatus* populations sampled from different lakes showed opposing trends in %P and N:P between sexes (Durston & El‐Sabaawi, 2017). Among mammals, differences in elemental composition related to sex arise mostly because of either parental care or mate search. Lactation and parental care exert costs due to increased foraging requirements in the parent administrating to the newborns, as is the case among small mammals such as the big brown bat *Eptesicus fuscus* (Hood, Oftedal, & Kunz, 2006). Similarly, the development of secondary sexual characteristics, for instance the yearly production of antlers in some ungulate species, dramatically increases the need of a few selected elements in one of the two sexes (Atwood & Weeks, 2002). While snowshoe hares are weakly sexually dimorphic (Feldhamer et al., 2003) and lack specialized secondary sexual characteristics, they can produce up to four litters per year, each comprising between 4 and 6 leverets. Females are larger than males, on average, likely as a strategy to offset this large reproductive investment (Feldhamer et al., 2003). Consequently, differences in the organismal content of C, N, or P could arise between sexes in hares following varying nutritional needs due to different reproductive strategies and efforts (Morehouse, Nakazawa, Booher, Jeyasingh, & Hall, 2010).

Organismal elemental composition can also vary with an individual's body size, as well as with its related condition metrics (body condition indexes, BCI; Stevenson & Woods, 2006). For instance, P content tends to scale with an organism's size, particularly among invertebrates (Back & King, 2013; González et al., 2011; but see Gillooly et al., 2005). While widespread, the sign of this relationship differs strongly among different groups, such as invertebrates and vertebrates. Among invertebrates, P content decreases with size, as they lack internal repositories of this element (González et al., 2011; Sterner & Elser, 2002). Conversely, among vertebrates the majority of P stocks are found in bone tissue, so the P‐body size allometric relationship should be positive (Sterner & Elser, 2002). That is, all else being equal, P concentration should increase as the body size of an individual increases. However, modeling approaches show that P content should initially decrease and eventually approach an asymptotic relationship with vertebrate body size (Gillooly et al., 2005). Yet, empirical evidence suggests vertebrates' organismal P content increases with body size: for instance, in the tropical stream fish *Rivulus hartii*, larger individuals have higher concentrations of P than their smaller conspecifics (El‐Sabaawi, Kohler, et al., 2012). Likewise, in the Atacama Desert of Chile, two species of lizards show a similar pattern of %P increasing with body size (González et al., 2011). In turn, this variability in the content of fundamental nutrients with body size could influence the overall condition of an individual—which ultimately determines its fitness and nutritional value for its predators (Stevenson & Woods, 2006). In a strongly N‐ and P‐limited environment like the boreal forest, snowshoe hares need access to large quantities of both N and P to develop muscle mass and skeleton over the course of a relatively short time (Pilati & Vanni, 2007). Thus, larger individuals could indeed show higher concentrations of N and P as they may prioritize or have easier access to these limiting nutrients over C, or other elements (Kay et al., 2005).

From all of the above it follows that, during an individual's lifetime, its content of C, N, and P likely varies as a result of age (Ebel et al., 2016), sex (Durston & El‐Sabaawi, 2017), or body size (El‐Sabaawi, Kohler, et al., 2012). Following previous works and theory on both consumer stoichiometry (Boros et al., 2015; Ebel et al., 2016; González et al., 2011) and snowshoe hare ecology (Krebs et al., 2018), we predict that (a) snowshoe hare organismal concentration of N and P increases as individuals grow older, whereas C concentration should decrease. We also expect (b) female hares to have higher overall concentration of N and P than males, due to higher reproductive costs, and lower C. Finally, we expect (c) larger snowshoe hares and those in better body condition to have higher concentrations of N and P. We provide predictions pertaining to the C:N, C:P, and N:P ratios in Appendix S1: section 1. We present one of the first assessments of the C, N, and P body composition of a terrestrial vertebrate and discuss how intraspecific stoichiometric variability might influence trophic dynamics and ecosystem processes.

## 2 METHODS

### 2.1 Study species

**Figure 1:**
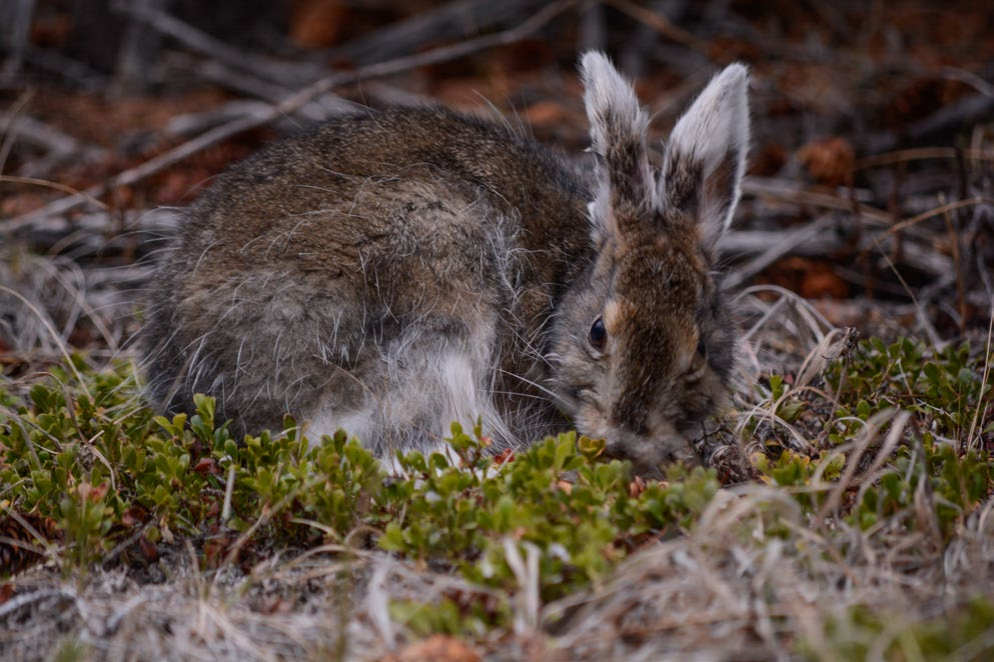
A snowshoe hare, *Lepus americanus*, in its summer livery. Photograph courtesy of Juliana Balluffi‐Fry

Snowshoe hares (Figure 1) are a keystone herbivore in the boreal forests of North America, with a geographic range extending from Alaska to New Mexico (Feldhamer et al., 2003). Average total body length of snowshoe hares varies between 36 and 52 cm and mean adult body weight is 1.3 kg (range: 0.9–2.3 kg): of this, only about 5% is fat, with both seasonal and annual fluctuations (Murray, 2002). Females are usually 10%–25% larger than males (Feldhamer et al., 2003).

Snowshoe hares are mostly nocturnal and do not hibernate over winter (Feldhamer et al., 2003). For these reasons, they are most often found in habitats with dense understory vegetation, allowing for more efficient thermo‐regulation and predator avoidance (Litvaitis, Sherburne, & Bissonette, 1985). Snowshoe hare populations cycle throughout the continent, with peaks every 8–11 years and densities ranging 5‐ to 25‐fold (Reynolds, Vander Wal, Adams, Curran, & Doucet, 2017). These abundance cycles are a defining characteristic of the boreal forest, affecting the ecology of many boreal species, from the plants the snowshoe hares consume, to their competitors and predators (Krebs et al., 2018).

Snowshoe hares were introduced in Newfoundland in 1864 and quickly spread across the island (Strong & Leroux, 2014). Studies conducted in the 1960s investigated their population dynamics, diet composition, and competition with another introduced herbivore, the moose *Alces alces* (Dodds, 1960, 1965). Compared with areas of Canada further west, Newfoundland has a fluctuating snowshoe hare population, with shorter and less regular periodicity (8–9 years; Reynolds et al., 2017). Their diet varies among seasons and areas of the island of Newfoundland (Dodds, 1960): black spruce *Picea mariana* and balsam fir *Abies balsamea* comprise most of the winter forage, whereas during the summer they forage almost exclusively on deciduous plants and shrubs (e.g., *Vaccinium* spp.; *Trifolium* spp.; *Viburnum* spp.; Dodds, 1960).

### 2.2 Data collection

#### 2.2.1 Snowshoe hare morphology, age, and sex

In October 2016, we purchased 50 whole, wild‐caught snowshoe hares from a local trapper and stored them in individual plastic bags at −20°C. These wild‐caught specimens came from four snaring locations in the Eastern Avalon peninsula, over a small 21.5 km^2^ trapping area around the towns of Chapel Arm (NL, 47°31′00″N, 53°40′00″W) and Long Harbour (NL, 47º25′46″N, 53°51′30″W). Individuals were snared without baits and thus likely reflect the age and sex distribution of the wild population they were taken from. In the laboratory, we thawed and weighed each specimen to the closest 0.1 g. We collected data on total body length, left hind foot length, and skull length and width for each hare to the closest mm, repeating each measurement three times and using their arithmetic mean in all subsequent analyses (see Appendix S1: section 2.1).

Like rodents, the teeth of lagomorphs grow continuously during their life, making conventional aging techniques based on dentine and cement inapplicable (Morris, 1972). To account for this, we aged our specimens using a mixed approach involving counting bone tissue growth lines deposited after each winter in the mandibular bone. We used an aging method developed for mountain hares *Lepus timidus* to select the area of the bone from which to count the growth lines (Iason, 1988). For all 50 snowshoe hares in our sample, we extracted the complete mandibular bone, cleaned it of all soft tissues, and shipped the clean bones to Matson's Laboratory (Manhattan, MT, USA) for age determination (see Appendix S1: section 2.2).

Sex determination in wild‐caught snowshoe can be difficult. While visual approaches are available, these can be challenging when used on young, not yet fully developed individuals (as was the case in this study, see Section 3 below). Genitalia are often not apparent in individuals younger than 6–12 months old and, even among not actively reproducing adults, they can be difficult to find. For these reasons, we chose to determine specimen sex using a DNA‐based approach (Shaw, Wilson, & White, 2003; see Appendix S1: section 2.3). As the snowshoe hare genome is not yet completely sequenced, we chose a widely used set of primers for genetic sex determination in mammals to amplify the genetic material extracted from our specimens and from two control snowshoe hares of known sex (Shaw et al., 2003). In cases when this DNA‐based approach failed to detect an individual's sex (*n* = 3), we determined it by visual inspection and palpation of the genital area.

#### 2.2.2 Body size metrics

We investigate the relationship between body size and organismal chemical composition of snowshoe hares using two different metrics: body condition and average body length. Body condition is a widely used metric to assess the overall health and quality, or “plumpness,” of animals (Peig & Green, 2010; Stevenson & Woods, 2006). Snowshoe hares, however, differ from other mammals in that they do not rely on fat tissue to store energy (see Section 2.1 above). Consequently, body condition indexes that rely on body fat content may not capture the real body condition of our sample of wild‐caught hares. For this reason, we estimate body condition using the scaled mass index (SMI; Peig & Green, 2009). The SMI standardizes an individual's measure of body size with respect to another, thus accounting for scaling relationships (Peig & Green, 2009). In particular, the SMI uses the average value of the length measurement (*L*) with the strongest relationship with its body weight (*M*) as the standardizing variable, as established by a Standardized Major Axis regression (Peig & Green, 2009; see Appendix S1: section 2.4). The SMI formula is:

(1) $${\hat{M}}_{i}=M_{i}{\left[\frac{L_{0}}{L_{i}}\right]}^{b_{\text{SMA}}}$$

where ${\hat{M}}_{i}$ is the SMI of individual *i*, *M_i_* is its body weight, *L_i_* is the linear measure of body size of *i*, *b*_SMA_ is the exponent (i.e., slope) of a Standardized Major Axis Regression of ln(*M*) over ln(*L*), and *L*_0_ is the study population's average value of *L_i_*. Therefore, the SMI is the expected weight of individual *i* if its length measurement *L_i_* was equal to the population's average value *L*_0_. In this study, we used the length of the left hind foot to calculate the SMI. From the SMI value, we then computed the relative body condition (*K*_n_) of an individual as the ratio of *M_i_* to ${\hat{M}}_{i}$ (Stevenson & Woods, 2006). This provided us with a simple metric to assess how good or bad an individual's condition was, compared with what it should be.

As the SMI is sensitive to the length measurement used to calculate it, we ran a separate set of models using a SMI produced using skull length, which also showed a strong relationship with body weight (see Appendix S1: section 2.4). Furthermore, we considered average body length as a separate estimate of the effect of body size on the C, N, and P stoichiometry of snowshoe hares (see above, Snowshoe hare morphology, age, and sex).

#### 2.2.3 Snowshoe hare C, N, and P body stoichiometry

After collecting both morphological data and bone samples required for aging, we individually blended our specimens to a homogeneous paste using a Retsch GM300 knife mill (Retsch GmbH). Through preliminary tests conducted on road‐killed individuals not included in our sample of 50, we noticed that elastic or fine tissues, such as fur, skin, ears, and the walls of the digestive tract, were particularly difficult to homogenize with our equipment. Consequently, we removed fur, skin, and ears from all specimens: as such, our definition of body here does not include fur, skin, and ears. For the digestive tract, instead, we removed, cleaned, and finely chopped it before adding it back into the mixture. For each specimen, we collected a sample of the homogenized mixture, weighed it for wet weight (g), and oven dried it to constant weight for an average of four nights at 50°C. After drying, we further ground each sample to as fine a powder as possible using mortar and pestle and weighed it again for dry weight (g). On average, we required 50 g of wet homogenized material to produce 10 g of dry material for determining element concentration. We transferred all ground samples to glass vials and stored them in desiccators to prevent moisture accumulation and mold formation.

We sent the 50 dried samples to the Agriculture and Food Laboratory (AFL) at the University of Guelph for determination of the body content of C, N, and P as % of each sample's dry weight. At AFL, each sample was further ground before stoichiometric analyses. Concentrations of C and N were obtained following standard practices with an Elementar Vario MACRO cube (Elementar Analysensysteme GmbH). For P, homogenized samples were first digested with nitric acid and hydrochloric acid using a closed‐vessel microwave (CEM Marsxpress; CEM Corporation). The microwave‐digested sample was then brought to volume with nanopure water and P content quantified using inductively coupled plasma‐optical emission spectroscopy using a Varian Vista Pro and a pneumatic nebulizer (Varian Inc.). The method was based on AOAC 2011.14.

Given that few studies have measured the C, N, and P body stoichiometry of terrestrial vertebrates, we ran pilot tests to assess within‐sample variability. These showed some within‐sample variability in %C and %N (Appendix S1: Figures S2 and S3). To account for this, each sample was analyzed three times for C and N content. Conversely, %P was relatively invariant within samples. Because of this, only five samples were run in duplicate to assess within‐sample variability in %P (see Appendix S1: section 2.5). In addition, to capture variability within individuals due to our homogenization protocol, we selected five random specimens for which we sent two additional samples (*n* = 10) of the homogenized paste to AFL (see Appendix S1: section 2.5). Upon receiving the results back from AFL, to obtain C, N, and P stoichiometry and molar ratios for each hare, we calculated each hare's dry body weight and converted the concentration of each element to molar mass using atomic weights. As variation among samples taken from each individual was negligible for all three elements, we used average values of %C, %N, and %P for each individual in subsequent analyses (see Appendix S1: section 2.6).

### 2.3 Statistical analyses

We used General Linear Models (GLMs) in R (v. 3.4.4; R Core Team, 2018) to investigate age, sex, body size, and condition as potential drivers of hare stoichiometry. We used the concentration of each element of interest (i.e., %C, %N, %P), as well as the ratios C:N, C:P, and N:P as our response variables. We chose to focus on both elemental concentrations and ratios as these different measurements convey different but complementary information on body composition: quantity of elements of interest and their relationship to each other and importance to the animal, respectively. Age (continuous), sex (categorical), relative body condition (*K*_n_, continuous), and average body length (ABL, continuous) were our explanatory variables. To test our predictions, we considered the effects of each of our predictor variables alone and their additive and 2‐way interactive effects. We tested for multicollinearity among our explanatory variables using variance inflation factor analysis (VIF). As expected, VIF showed that relative body condition and average body length were highly correlated (VIF > 3). Therefore, we did not include these two variables in the same model (see Appendix S1: section 3). We fit a set of 22 competing models, including an intercept‐only model and used the function AICc from the AICmodavg R package to select the most parsimonious model based on the Akaike Information Criterion corrected for small sample size (AICc; Burnham & Anderson, 2002; Mazerolle, 2019). We then removed models with uninformative parameters (Leroux, 2019) from the model set of each response variable (Leroux, 2019; see Appendix S1: section 4.1).

## 3 RESULTS

Snowshoe hares in our sample varied in age between 0 (“young‐of‐the‐year”) and 6 years old, the majority (74%) being between 0 and 1 years old. Only one individual, a female, was 6 years old. Males were more common (31 out of 50) than females (19). Average (±*SD*) wet body weight was 1,374.81 g (±186.59, range: 914.30–1,776.50 g), with average dry weight being 399.11 g (±74.70, range: 241.76–567.86 g). Water made up to 72% of body weight. Average body length was 42.49 cm (±2.07, range: 36.67–46.67 cm; Table S16). Average left hind foot length (*L*_0_) for our snowshoe hare population was 12.88 cm (±0.58, range: 11.40–14.10 cm). The slope of the Standardized Major Axis regression of average left hind foot length on body weight (i.e., the exponent *b*_SMA_ in Equation 1) was 3.18. Overall, young snowshoe hares appeared more variable in relative body condition than older individuals (mean: 1.01 ± 0.14; Figure S5).

**Figure 2:**
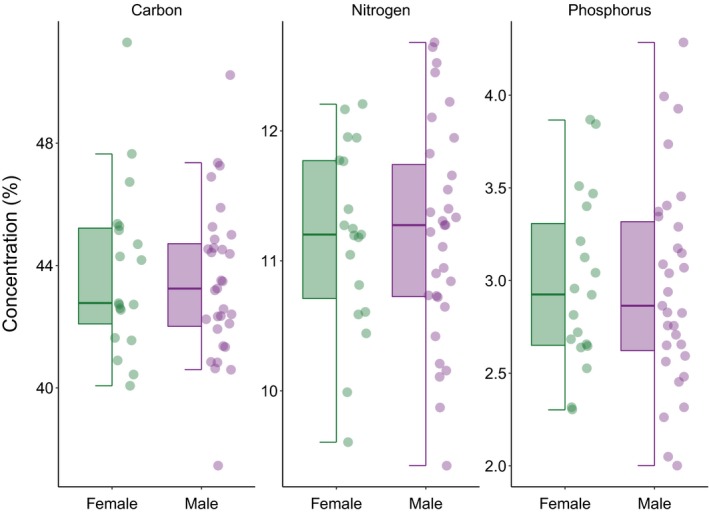
Sex‐related variability in the concentrations of carbon (C), nitrogen (N), and phosphorus (P) among 50 snowshoe hares. The lower and upper boundaries of the box are the first and third quartiles, respectively. The thick horizontal line inside the box is the median, that is, the second quartile. The whiskers extend from either boundary to no further than the largest (or smallest) value * 1.5 IQR (interquartile range). Female snowshoe hares show higher median values of %P than males. Males, on the other hand, appear consistently more variable than females in their content of both N and P. Note the different scales of the *y*‐axis among the three panels

**Figure 3:**
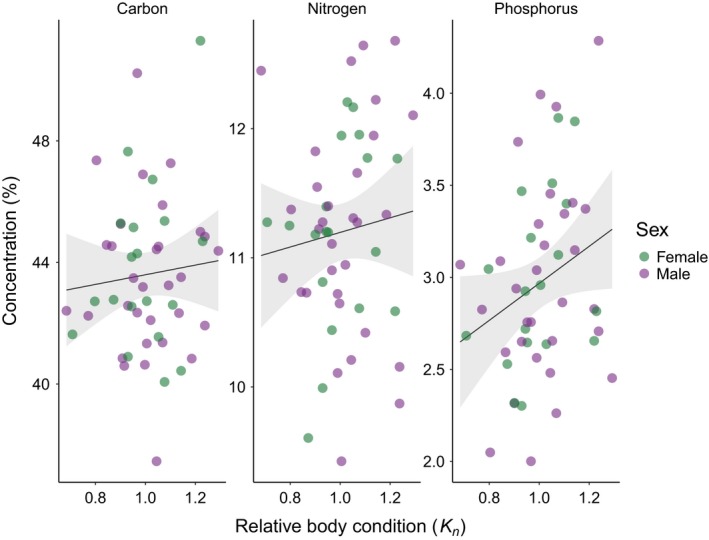
Variability in the concentrations of C, N, and P with increasing relative body condition. The positive trend for P is evident and is weakly supported by the results of our modeling. Conversely, there is no visual evidence of a relationship between %C or %N and relative body condition, which is further confirmed by the results of our modeling (Table 1). Solid lines are ordinary least square regression lines, shaded areas represent 95% confidence intervals around them

**Figure 4:**
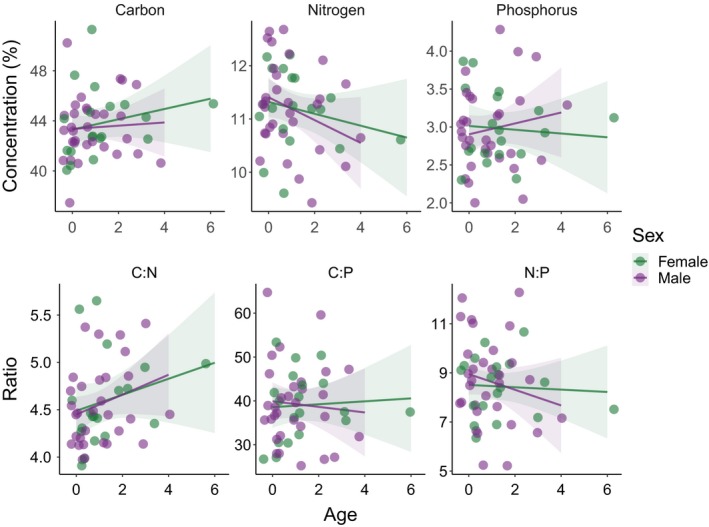
Variability in C, N, and P concentrations and their stoichiometric ratios with increasing age among 50 snowshoe hares. Upper panels: while concentrations of P appear largely invariant as age increases, we notice a negative trend for N concentration for both sexes. This is further supported by the weak relationship found between age and %N through our modeling approach. Lower panels: values of C:N appear to increase with age, regardless of sex, as would be expected given the negative relationship between %N and age. Conversely, the values of N:P seem to decrease as males get older. No trend is evident for C:P, which is in line with the lack of pattern in the variability of %C. We added a jitter of 0.4 to both axes to improve readability of the graphs. All other specifications as in Figure 3

Snowshoe hares were, on average, composed of 43.60% C (±2.59, range: 37.46%–51.29%), 11.20% N (±0.78, range: 9.42%–12.68%), and 2.97% P (±0.52, range: 2.00%–4.29%; Figure 2 and Table S17). The most parsimonious model for %N included only age (*R* ^2^ = 0.066): %N was negatively related to the age of individual snowshoe hares (Table 1). Evidence for this relationship is, however, weak as the intercept‐only model was within 2 ΔAICc of the top‐ranked model (Table 1). For %P, the two top‐ranked models included relative body condition and average body length, respectively (Table 1). %P was positively related to relative body condition (*R* ^2^ = 0.073; Figure 3) and average body length (*R* ^2^ = 0.047). Again, evidence for these relationships is weak as the intercept‐only model was the third best‐performing model and within 2 ΔAICc of the top‐ranked models (Table 1). We also observed a qualitative pattern of higher %P among older males (Figure 4), but found no statistical support for it (Table 1). For %C, the top‐ranked model was the intercept‐only model, which provides evidence for no relationship between variation in %C and age, sex, or body size and condition of individuals (Table 1).

**Table 1:** Top ranking GLMs for %C, %N, and %P based on ∆AICc values. We report only models that ranked better than the null model, together with the null model. *k*, number of parameters in the model; LL, log‐likelihood; *K*_n_, relative body condition; ABL, average body length. We provide coefficient values as estimate (±*SE*)

*k*	LL	ΔAICc	*R* ^2^	Coefficients			
				Intercept	Age	*K*_n_	ABL
%N top models							
3	−56.599	0.000	.066	11.367 (±0.141)	−0.160 (±0.087)		
2	−58.306	1.150	.000	11.200 (±0.111)			
%P top models							
3	−35.556	0.000	.073	1.962 (±0.526)		1.006 (±0.518)	
3	−36.252	1.391	.047	0.687 (±1.495)			0.054 (±0.035)
2	−37.444	1.508	.000	2.974 (±0.073)			
%C top models							
2	−118.09	0.000	.000	43.606 (±0.367)			

For stoichiometric ratios, the top‐ranked model for C:N included only age, which had a positive relationship with C:N ratio (*R* ^2^ = 0.074; Table 2). For this relationship too, evidence is weak as the intercept‐only model was within 2 ΔAICc of the best‐performing one. We found no evidence for a relationship between age, sex, body size, and condition, and either C:P or N:P as the top‐ranked model for both these ratios was the intercept‐only model (Table 2). Using skull length instead of left hind foot length to calculate *K*_n_ did not qualitatively change our results (see Appendix S1: Tables S1 and S2).

**Table 2:** Top ranking GLMs for C:N, C:P, and N:P based on ∆AICc values. All specifications as in Table 1

*k*	LL	ΔAICc	*R* ^2^	Coefficients			
				Intercept	Age	*K*_n_	ABL
C:N top models							
3	−27.818	0.000	.074	4.465 (±0.079)	0.095 (±0.049)		
2	−29.731	1.559	.000	4.564 (±0.063)			
C:P top models							
2	−178.30	0.000	.000	39.205 (±1.223)			
N:P top models							
2	−94.153	0.000	.000	8.58 (±0.227)			

## 4 DISCUSSION

We provide one of few assessments of the C, N, and P body stoichiometry of a terrestrial vertebrate and investigate potential drivers of this fundamental ecological trait. Overall, we find considerable variation in the concentrations of C, N, and P, and in their ratios within our sample of snowshoe hares. However, age, sex, and body size or condition provide little or no explanation of this variation. Our models highlight a weak negative relationship between an individual's age and its N concentration and, symmetrically, a weak and positive trend of C:N and age. Likewise, we find weak support for a relationship between an individual's body size and condition and its P concentration. Together, these results provide some of the first evidence for intraspecific variability in the C, N, and P body stoichiometry of a terrestrial vertebrate but raise the need to consider a broader suite of potential drivers. As well, our data provide a starting point for comparisons of vertebrate species stoichiometry across ecological realms (e.g., aquatic‐terrestrial).

We found weak evidence in support of our prediction that age might drive variability in the C, N, and P body stoichiometry of snowshoe hares. In particular, contrary to our predictions, we find weak evidence of young individuals (0–1 years old) having higher N concentrations than older ones—with a more pronounced decrease among males than among females (Figure 4). As would be expected from this pattern, C:N values show an opposite, positive trend with age (Figure 4)—reflecting the lower amounts of N compared with C in older hares and lending further support to this result. Younger individuals may show higher %N as a result of increased N allocation to muscle tissue production (Boros et al., 2015). Snowshoe hares experience strong predation pressure from a large cohort of predators, both land‐based and avian, from the earliest life stages (Krebs et al., 2018). A higher N content among young hares could be a sign of early‐life investments in production of N‐rich protein to develop the muscle mass necessary for their hide and run anti‐predator response. We also observed a qualitative pattern of increasing %P with age among males. While our models do not offer quantitative support for it (Table 1), similar patterns have been described for other vertebrate species. Boros et al. (2015) found a similar trend between %P and age in two species of laboratory‐reared minnows. Similarly, Sterrett, Maerz, and Katz (2015) found that older individuals had higher %P in four species of turtles. This pattern could result from bone tissue development as the individual ages (Sterner & Elser, 2002). In turn, by actively sequestering P in their bones, vertebrates could influence nutrient ecosystem dynamics by acting as “walking” repositories of a limiting element (Pastor et al., 2006; Sterrett et al., 2015). Given the large number of young individuals in our sample, and the relative rarity of hares older than 3 years, it may be that access to P during a hare's aging process is fundamental for its survival. Future studies investigating the link between N and P availability and long‐term survival in wild herbivore populations may further our understand of both population dynamics and ecosystem impacts mediated by these consumers.

Contrary to our predictions, we find no evidence for a relationship between hare stoichiometry and sex. Male individuals did show larger variability in their N and P concentration than females (Figure 2) but our models provide no quantitative support for a relationship between sex and body stoichiometry. This may not be surprising given the low sexual dimorphism shown by our study species. Several studies that investigated the relationship between sex and organismal stoichiometry among more strongly sexually dimorphic species provide similarly contradictory evidence. Among guppies, for instance, sex had no relationship with stoichiometry when considered alone, yet it had significant interactions with the fish's stream of origin—likely an indirect consequence of different predation levels experienced by males and females in different streams (El‐Sabaawi, Zandonà, et al., 2012). Conversely, among *Hyalella* amphipods, strong sexual dimorphism in the concentrations and patterns of variation of multiple elements underlays sexual dimorphism in traits as different as foraging behavior, nutritional physiology, and sex‐specific selection of genomic loci (Goos, Cothran, & Jeyasingh, 2017). Among antler‐producing ungulates, males and females differ in both content and use of certain elements (e.g., calcium; Atwood & Weeks, 2002). Finally, as hares undergo morpho‐physiological changes during their reproductive season, investigating the relationship between C, N, and P body stoichiometry and sex among actively reproducing hares might produce different results (Hood et al., 2006). These contrasting lines of evidence highlight the need of further research, involving a wider range of species from a variety of environments, to reduce the uncertainty around the role of sex as a driver of variation in organismal stoichiometry.

Consistent with our predictions, our results indicate body size and condition as potential drivers for variability in P concentration in our sample. The two top models for this element included relative body condition and average body length, and both variables had a positive relationship with %P. In particular, the observed body weight of snowshoe hares with higher %P matched or exceeded the predicted value obtained from the SMI formula (Equation 1). Snowshoe hare body condition fluctuates throughout the year (Murray, 2002), with peaks in the months leading up to the boreal winter, during which hares remain active and face increased levels of stress due to both lack of optimal forage and increased predation (Krebs et al., 2018). As body condition declines over the winter months (Murray, 2002), one could test if the weak relationship we observe between P and body condition would vary in a similar way. Additionally, we observe a qualitatively larger variability in relative body condition among young hares in our sample than among older specimens (Figure S5). Snowshoe hares produce multiple litters per year (up to four; Feldhamer et al., 2003), yet a large number of leverets does not survive the first winter (Krebs et al., 2018). While we do not find evidence for a relationship between age and P content, a potential question to ask is whether birth date within a year could explain part of this variability. Our results, albeit weakly supported by our statistical analyses, appear to confirm the potential role P plays within the internal chemical machinery of an animal, and its importance for its survival (Boersma et al., 2008; Elser et al., 2007).

A large amount of variability in our sample remains unexplained and, overall, we find only weak support for our initial hypothesis of variation in organismal stoichiometry among snowshoe hares. Indeed, other vertebrate species show much stronger patterns of intraspecific variation in elemental content. Ebel et al. (2015), Ebel et al. (2016), for instance, showed that migratory Atlantic salmon *S. salar* at different ontogenic stages have distinct stoichiometric signatures, particularly before and after their first migration from their freshwater nurseries to the open ocean. The reason for these differences in the magnitude of the effects mediated by ontogeny could be found in the life history of snowshoe hares. Snowshoe hares do not undergo dramatic life events like migratory salmon, or the metamorphosis of certain insect species, which clearly separate different life stages. Rather, they are characterized by short gestation periods (≃30–40 days) and quick maturation of leverets into adults (≃6 months; Feldhamer et al., 2003). It is possible, in this scenario, that we investigated the effects of age at a time in the life of snowshoe hares when most of the changes in chemical composition had already taken place. It is also interesting to note the larger proportion of young individuals in our sample, consistent with current knowledge about snowshoe hare survival beyond their first winter (Krebs et al., 2018) and likely representative of the age distribution of the particular wild population we used in this study. Thus, a potentially interesting and rewarding research avenue would be to further investigate differences in hare stoichiometry in earlier life stages.

Before homogenization, we removed fur, skin, and ears of our snowshoe hare specimens, as these tissues proved challenging to homogenize. While we consistently applied this protocol to all 50 hares included in our sample, excluding these tissues from analyses may have influenced the amount of stoichiometric variability we detected. Ears are made of cartilage, which consists mostly of polysaccharides and proteoglycans, thus being C‐rich. Additionally, on average, ears accounted for 0.74% of a hare's wet body weight in our sample. As for fur and skin, in humans and other mammalian species, hair is made of up to ~17% N (Block, Bolling, Brand, & Schein, 1939). While we could not find accounts of the chemical composition of snowshoe hare hair, it is possible that the production, maintenance, and molting processes of this species' fur impose further stoichiometric requirements—ultimately influencing the relative concentration of C, N, and P in a snowshoe hare body throughout its life. Finally, although our samples were collected from a small area, fine scale forage quality may also be a driver of the stoichiometric variability we observed. As well, snowshoe hare populations from different areas of Newfoundland and North America may differ in their elemental composition from the specimens investigated here (as is the case for some fish species; El‐Sabaawi, Zandonà, et al., 2012). Future work could investigate spatial variation in habitat and forage quality as a driver of consumer body elemental composition (Leroux et al., 2017), and interpopulation variability.

The variation in hare body composition we observe could have repercussions beyond the stoichiometry of this species and influence ecosystem processes such as nutrient cycling, transport, and primary productivity (Pastor et al., 2006). Snowshoe hares are a keystone herbivore in the boreal forest, a markedly nutrient‐limited environment (Pastor et al., 2006). They are characterized by strong, decade‐long fluctuations in their population abundance and serve as primary food source for multiple predator species (Krebs et al., 2018). Paucity of nutrients, and the well‐known stoichiometric mismatch between plants and herbivores (Elser, O'Brien, Dobberfuhl, & Dowling, 2000; Sterner & Elser, 2002), prompted boreal forest herbivores to evolve browsing strategies allowing them to extract as many nutrients as possible from their food (Pastor et al., 2006). Thus, the appearance of a large number of young snowshoe hares over the landscape during a population peak could have strong dampening effects on elemental cycling in the boreal forest—as well as in adjacent ecosystems—possibly reducing N or P availability to primary producers as they become locked within the herbivores' biomass. By infusing ongoing ecological research with stoichiometric data, future studies could address this potential interplay between a species' stoichiometry and the ecosystem processes it contributes to (Leal et al., 2017). In turn, this would allow for shedding light on fine‐grain mechanisms with far‐reaching consequences, such as cross‐ecosystem nutrient mobilization (Schmitz et al., 2018) and nutrient recycling (Schmitz et al., 2014), as well as on their influence on ecosystem services fundamental for humans.

Ecological stoichiometry has a long history in marine and freshwater ecosystems and has been shaped by detailed studies of algae, plants, and invertebrates. In recent years, researchers started investigating the stoichiometry of more complex organisms in aquatic ecosystems, particularly fish (Atkinson, Capps, Rugenski, & Vanni, 2017). This expanded the reach of ecological stoichiometry in exciting new directions, integrating it with other subfields of ecology, such as metabolic ecology (Rivas‐Ubach et al., 2012), ecosystem ecology (Abbas et al., 2012), and landscape ecology (Leroux et al., 2017; Sardans et al., 2016). Yet, terrestrial species other than plants and insects remain relatively unexplored in terms of their stoichiometry. Our results suggest that a greater focus on terrestrial vertebrates and consumers could provide novel insights and potentially question well‐known concepts in this field.

## CONFLICT OF INTEREST

None declared.

## AUTHOR CONTRIBUTIONS

MR, SJL, EVW, and YFW devised the study; MR, TRH, JBF, SJL, YFW, and EVW collected the data; MR and SJL analyzed the data; MR, TRH, JBF, SJL, YFW, and EVW interpreted the data; MR led the writing of the manuscript. All authors contributed critically to the drafts and gave final approval for publication.

### Open Research Badges

This article has earned an https://openscience.com and https://openscience.com Badges for making publicly available the components of the research methodology needed to reproduce the reported procedure and analysis. All materials are available at [https://doi.org/10.6084/m9.figshare.7884854.v2].

## Data Availability

Data and code used in the analyses are available via the figshare online repository at: https://doi.org/10.6084/m9.figshare.7884854.v2
